# Discovery of an underground chamber to protect kings and queens during winter in temperate termites

**DOI:** 10.1038/s41598-023-36035-1

**Published:** 2023-05-31

**Authors:** Mamoru Takata, Takao Konishi, Shuya Nagai, Yao Wu, Tomonari Nozaki, Eisuke Tasaki, Kenji Matsuura

**Affiliations:** 1 Laboratory of Insect Ecology, Graduate School of Agriculture, Kyoto University, Kitashirakawa-Oiwake-cho, Sakyo-ku, Kyoto, 606-8502 Japan; 2 Department of Biology, Faculty of Science, Niigata University, 8050 Ikarashi 2-no-cho, Nishi-ku, Niigata, 950-2181 Japan

**Keywords:** Behavioural ecology, Evolutionary ecology

## Abstract

Overwintering is a critical part of the annual cycle for species that live in temperate, polar, and alpine regions. Consequently, low-temperature biology is a key determinant of temperate species distribution. Termites are distributed predominantly in tropical regions, and a limited number of species are found in the temperate zone. Here, in the termite *Reticulitermes speratus*, we report the discovery of an underground chamber that protects kings and queens to survive the winter, which is separate from the one they used during the warmer breeding season. In the spring, the royals inhabited decayed logs on the ground, then moved to their underground chamber located in the roots of stumps in the fall. The winter minimum temperature measured in the royal chamber was higher than that in the logs on the ground. In overwintering termites, the kings and queens had higher cold tolerance than workers and soldiers. Air temperatures dropped below the critical temperature multiple times, as evidenced from the past 140 years of weather records in Kyoto. These results demonstrated the survival strategies of reproductives to overcome the environment at the latitudinal limits. This study helps further the understanding of the termite’s seasonal phenology, long-term survivorship, and life cycle.

## Introduction

Temperature is a major factor restricting the distribution of almost all organisms^1^. Insects are susceptible to fluctuations in temperature^2^, and the majority of their activity is limited by the low temperatures in temperate, polar, or alpine regions. Insects acquired a variety of behavioural (heat and/or cold avoidance, temporal activity, etc.) and physiological mechanisms (production of antioxidants, antifreeze proteins, cryoprotectants, etc.) to survive under extreme temperatures^3–8^. Therefore, understanding the behavioural and physiological mechanisms which contribute to improving their persistence and the temperature at which they are at risk of mortality is fundamental to predicting their geographic distribution, resulting from climate change, and population dynamics, as well as seasonal phenology, long-term survivorship, and life cycle.

Social insects establish a well-organized society that is characterized by the reproductive division of labour among castes^9,10^. Reproductive castes predominantly produce the colony members such as workers and soldiers^11,12^. Therefore, the survival of reproductives is crucial for termites to maintain a thriving society. Kings and queens are generally protected by social-level defences provided by non-reproductives, which greatly reduces the risk of extrinsic mortality by predation, disease, starvation, desiccation, and extreme temperatures^13,14^. The elaborate nest structure is one of the most essential components of their social defence, and enables them to expand habitats by insulating extreme temperatures to create favourable microenvironments^15,16^. Thus, the location of the reproductives when temperatures are unsuitable for survival is key for ultimately understanding how colonies survive at the latitudinal limits.

**Figure 1:**
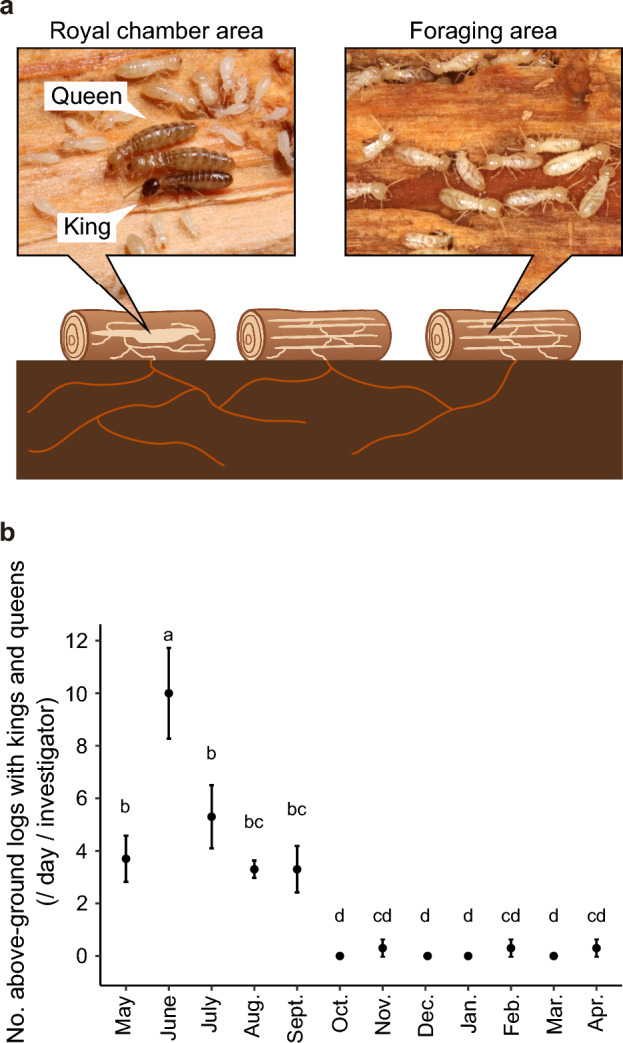
Seasonal location of kings and queens of the termite *Reticulitermes speratus*. (**a**) Diagram of multiple-site nesting. Single colonies use multiple wood sources and the nests are connected by underground tunnels. Royal chamber and foraging areas refer to nests in decayed logs with or without the king and queen, respectively. (**b**) Seasonal prevalence of kings and queens in logs on the ground. Different letters denote significant differences at *P* < 0.05, GLMM followed by a Tukey multiple comparison test.

Termites are generally tropical insects, but some species have adapted and distributed across temperate zone^17–20^. Winter temperature is the primary environmental factor that limits the distribution of termites at high altitudes^21^. In the northern hemisphere, *Reticulitermes* species live mostly in temperate forests.* R. speratus* is one of the best-studied termite species in terms of reproductive system^22^. It lives mostly in oak/pine mixed forests ranging from Kyushu to Hokkaido in Japan^23,24^. A single colony uses multiple wood types (fallen logs, stumps, etc.) which are connected by underground tunnels (Fig. 1a)^25,26^. Mature natural colonies contain more than 100,000 workers^27^, and are typically headed by one primary king and multiple secondary queens which are produced by parthenogenesis and replaced the primary queen^22,28,29^. Since it takes more than five years for a colony to start producing secondary queens and fertile dispersers, alates^28^, kings and queens have to survive multiple winters. In warm seasons, from May to October, the kings and queens are protected and cared for in chambers located deep inside the logs on the ground^30,31^. Termite kings and queens are extremely cryptic due to their multiple-site nesting behaviour and their high level of social defence. As a result, little is known about their overwintering biology and behaviour.

Herein we investigated seasonal movement of the royals and their cold tolerance in *R. speratus*. We first inspected decayed logs on the ground throughout the season to detect the seasonal movement of the royals. Second, after noticing kings and queens were missing almost entirely from the logs in winter, we traced their tunnels and located the underground chambers. Third, since the royals were found in the roots of stumps in winter, we investigated the above-ground and underground parts of stumps in summer to demonstrate that they are in the above-ground chambers during warm seasons and move underground when it gets colder. Forth, we used data loggers to investigate the winter minimum temperature in the underground royal chambers and the chambers in the logs above the ground, as well as the air near the ground surface. Fifth, we conducted a laboratory experiment to analyse the cold tolerance of each caste in overwintering termites. Finally, to assess the risk of mortality for kings and queens due to low temperatures when overwintering in above-ground logs, we also obtained air temperature data from the Japan Meteorological Agency^32^.

## Results

### Seasonality in the location of royal chambers

**Figure 2:**
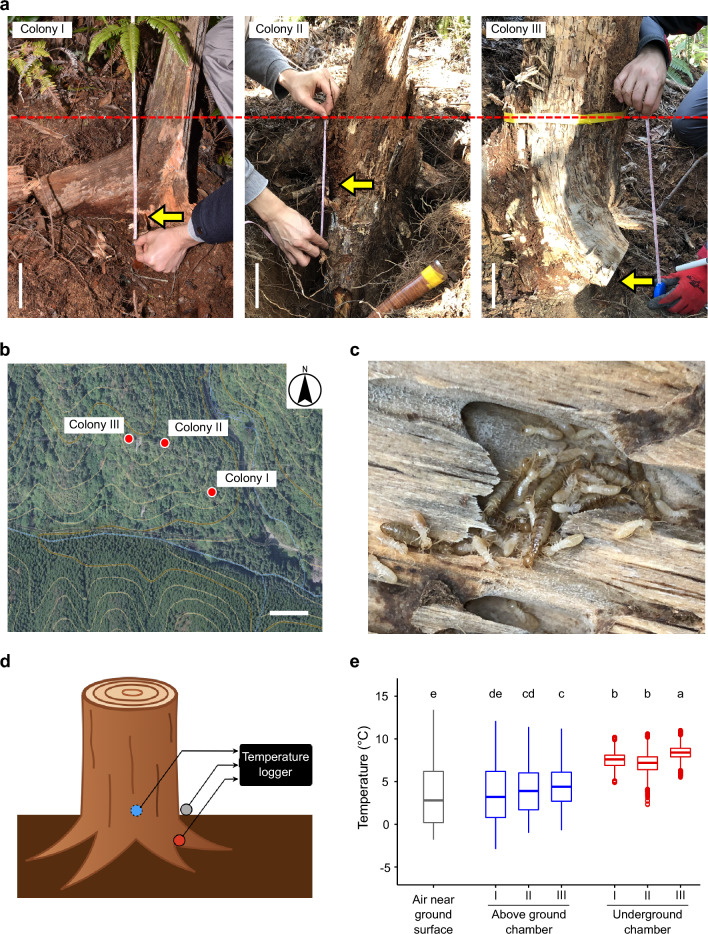
Cold avoidance by kings and queens of the termite *Reticulitermes speratus*. (**a**) Depth of underground winter royal chambers. Yellow arrows indicate the place where royals were found. The dashed red line shows ground level. White bars indicate 10 cm. (**b**) Location of the colony with winter royal chambers. A map and photograph of the forest in Yamashina-ku, Kyoto, Japan are provided by the Geospatial Information Authority of Japan. Red circles denote the location of each colony. The white bar indicates 50 m. (**c**) Photo of winter royal chamber. (**d**) Side view of temperature logging setup. Temperature probes were placed in the underground royal chamber (red circle), in the above ground chamber (blue circle), and in the air near the ground surface (grey circle). (**e**) Comparison of temperatures among the air near the ground surface, the above ground chamber, and the underground royal chamber during the coldest month (January). Temperature data were recorded hourly in three colonies (colony I, II, and III). The temperature in the air near the ground surface, the above ground chamber, and underground royal chamber are shown in grey, blue, and red, respectively. Different letters represent significant difference *P* < 0.05, paired *t* test with Bonferroni correction.

Our field survey revealed that the location of the kings and queens changes seasonally (Fig. 1, GLMM followed by Tukey multiple comparison tests, *P* < 0.001). From May to September which are warm season in studied areas, the royals are in the decayed logs on the ground. By contrast, from October to April which are cold season in studied area, they disappeared from the logs on the ground, and it was almost impossible to spot them from the logs above ground (Fig. 1b and Table 1). By tracing the tunnels starting in the logs on the ground surface, we located underground winter royal chambers in three colonies (Fig. 2a). These colonies were located on the southward facing side of the mountain slope (Fig. 2b). The elevation of each colony was 166, 190, and 196 m from the sea level. The mountain had the extensive tree cover and the forest floor had many decayed logs and dense fern growth. The soil could be classified as loamy and uniform in size, while slightly damp and permeable. The winter royal chambers were in the roots of stumps at 15, 24, and 37 cm underground (Fig. 2a). Workers, soldiers, and larvae were also found in the roots of the stumps in addition to the royals. Nymphs were found only in colony III. In colony I and II, no individuals were in the above-ground part of the stump. In colony III, although most individuals were found in the underground part of the stump, some workers and larvae were also observed within 5 cm of the ground surface. The termites were completely motionless when we opened the chambers irrespective of the caste and colony (Fig. 2c). In contrast to the winter season, the royal chambers in the summer season were found only in above-ground logs. All the colonies found in our survey included multiple secondary queens.

**Table 1:** Seasonal changes in total number of the colonies with royals found per investigating event per investigator. Decayed logs on the ground were examined for termite kings and queens by three investigators (KM, MT, and ET) once a month. Total number of the colonies with kings and secondary queens found per investigating event per investigator are shown.

Month	Location	Elevation range (m)	No. of colonies with royal chambers found		
			KM	MT	ET
May	Ukyo-ku, Kyoto, Japan	339–360	2	4	5
June	Soraku, Kyoto, Japan, and Nara, Nara, Japan	196–428	7	13	10
July	Nantan, Kyoto, Japan	364–438	3	7	6
August	Himeji, Hyogo, Japan	139–246	3	4	3
September	Tambasasayama, Hyogo, Japan	151–263	2	5	3
October	Yamashina-ku, Kyoto, Japan	212–230	0	0	0
November	Obama, Fukui, Japan	143–338	1	0	0
December	Yamashina-ku, Kyoto, Japan	124–312	0	0	0
January	Kita-ku, Kyoto, Japan	360–523	0	0	0
February	Ukyo-ku, Kyoto, Japan	299–363	0	1	0
March	Sakyo-ku, Ukyo-ku, Yamashina-ku, and Kita-ku, Kyoto, Japan	151–434	0	0	0
April	Yamashina-ku, Kyoto, Japan	217–263	1	0	0

### Temperatures in the winter royal chamber and above ground

In the coldest season in the study area, January, the temperature in the underground winter royal chambers was on average 3.0–3.8 °C higher than that in the chambers in the logs above the ground (two-tailed paired *t* test with Bonferroni correction, colony I: *t* = 38.459, *df* = 743, *P* < 0.001; colony II: *t* = 43.836, *df* = 743, *P* < 0.001; colony III: *t* = 57.590, *df* = 743, *P* < 0.001) and 3.5–4.7 °C higher than in the air near the ground surface (two-tailed paired *t* test with Bonferroni correction, colony I: *t* = 34.691, *df* = 743, *P* < 0.001; colony II: *t* = 32.348, *df* = 743, *P* < 0.001; colony III: *t* = 42.352, *df* = 743, *P* < 0.001), respectively (Fig. 2e). The lowest temperatures in the winter royal chambers and the logs above the ground were on average 4.2 °C and − 1.5 °C, respectively. The lowest winter temperature in the air near the ground surface was − 1.8 °C. As a result of the logs and soil acting as a buffer, the lowest temperatures in the winter royal chambers and the logs above the ground were 6.0 °C and 0.3 °C higher than the ambient temperature, respectively. The temperature was significantly more stable in the royal chamber than that in the chambers in the logs above the ground (*F*-test, colony I: *F*_743, 743_ = 6.303, *P* < 0.001; colony II: *F*_743, 743_ = 3.081, *P* < 0.001; colony III: *F*_743, 743_ = 4.354, *P* < 0.001), and in the air near the ground surface (*F*-test, colony I: *F*_743, 743_ = 7.078, *P* < 0.001; colony II: *F*_743, 743_ = 4.565, *P* < 0.001; colony III: *F*_743, 743_ = 7.766, *P* < 0.001).

### Lower lethal temperatures in each caste

**Figure 3:**
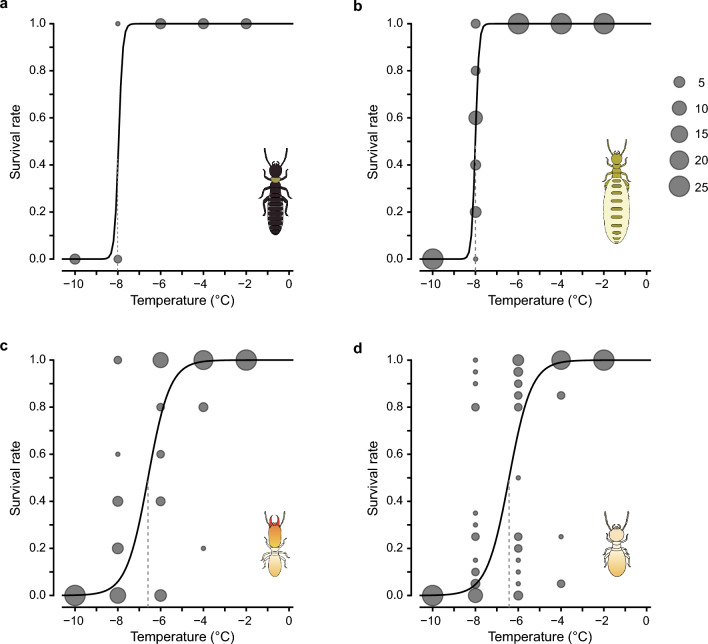
Comparison of the cold tolerance among castes in the termite *Reticulitermes speratus*. Survival after exposure to sub-zero temperatures in overwintering (**a**) primary kings, (**b**) secondary queens, (**c**) soldiers, and (**d**) workers. Survival rate of each colony at each test temperature are presented. The size of the circle represents the number of colonies. Solid curves and vertical dashed lines indicate fitted logistic curves and estimated lower lethal temperature of 50% of the population, respectively.

There were significant differences in cold tolerance among castes (Fig. 3, GLM, *χ*^2^ = 215.29, *df* = 3, *P* < 0.001). The kings and queens had significantly higher cold tolerances than workers and soldiers (GLM pairwise comparison with Holm correction, *P* < 0.001). The lower lethal temperature of 50% of the population in kings, queens, workers, and soldiers were estimated at − 8.0, − 8.0, − 6.6, and − 6.4 °C, respectively. A sudden increase in mortality began at − 8 °C for the kings and queens, and at − 4 °C for the workers and soldiers. Thus, if they overwintered in the underground royal chambers and above-ground logs, taking into account the 6.0 °C and 0.3 °C buffering effect of each chamber, they would be exposed to a considerable risk of mortality when the ambient temperature drops below − 14.0 °C and − 8.3 °C, respectively.

### Temperature records in Kyoto city

**Figure 4:**
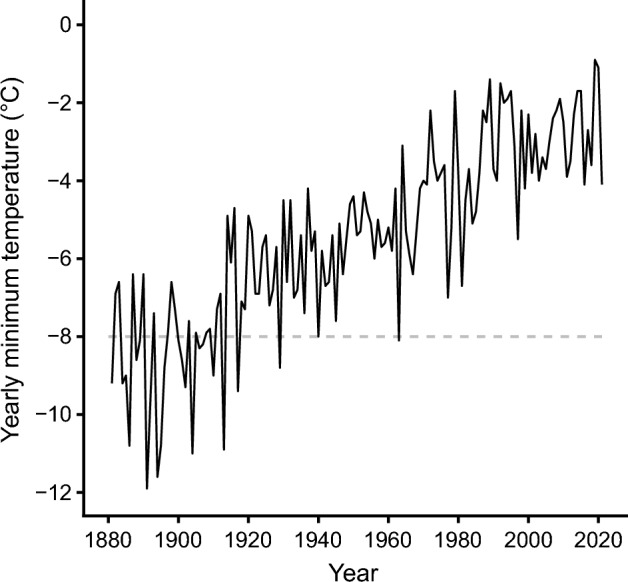
Yearly minimum temperatures in Kyoto, Japan from the past 140 years. The horizontal grey dashed line indicates the temperature at which kings and queens of the termite *Reticulitermes speratus* face the risk of mortality. The temperature data were obtained from the Japan Meteorological Agency (http://www.jma.go.jp/jma/indexe.html).

The meteorological data in the past 140 years in Kyoto showed that the temperature never fell below − 14.0 °C and the lowest recorded temperature in Kyoto was − 11.9 °C. There have been 18 years with at least one day when the annual minimum ambient temperature was below − 8.3 °C in the last 140 years in Kyoto (Fig. 4). Due to recent climate change, the last record of such an instance was from 1963.

## Discussion

Social insects have acquired a variety of traits that serve to prevent mortality of kings and queens induced by predators as well as harsh environments^13,14^. Termites are generally found in tropical regions as the temperate zone is their latitudinal limit of distribution^17–20^. The kings and queens have to survive more than five winters for the colony size to become large enough to produce alates^28^. Thus, kings and queens of temperate termites are expected to have traits that contribute to preventing cold-induced mortality. Here, we revealed the behavioural and physiological traits the termite *R. speratus* uses to overcome cold environments. Termites had an underground chamber for their kings and queens to get through the winter. The chamber was separate from the one they use during the warm breeding season (Figs. 1, 2 and Table 2). In the egg-producing seasons, from May to September^33,34^, the royals were in decayed logs on the ground (Fig. 1). When the temperature begins to drop, however, they almost entirely moved to underground royal chambers located in the roots of stumps (Fig. 2a,c). Here, temperatures were considerably warmer and more stable than that on the ground surface due to the soil acting as a buffer (Fig. 2e). In addition to the cold avoidance, cold tolerance in the kings and queens was higher than in workers and soldiers (Fig. 3). The meteorological data suggest the combination of the cold avoidance and tolerance has allowed the royals to survive the coldest winter in the past 140 years in Kyoto (Fig. 4). The identification of winter royal chambers and determination of lethal low temperatures in the kings and queens clearly show key traits to avoid the risk of overwintering mortality in temperate termites.

**Table 2:** Location of royal chambers in the summer. Stumps were dug out and examined for termite kings and queens in summer. The location (above or below ground) of royal chamber, number of primary king (PK), secondary king (SK), primary queen (PQ) and secondary queen (SQ) are shown.

Date	Location	Elevation (m)	Location of royal chamber	No. of individuals			
				PK	SK	PQ	SQ
2021/5/27	Takashima, Shiga, Japan	600	Above ground	1	0	0	35
2021/6/5	Tamba, Hyogo, Japan	261	Above ground	1	0	0	32
2021/6/5	Tamba, Hyogo, Japan	263	Above ground	1	0	0	42
2021/6/18	Miegunkomono, Mie, Japan	230	Above ground	1	0	0	33
2021/6/18	Miegunkomono, Mie, Japan	236	Above ground	1	0	0	97
2021/6/18	Miegunkomono, Mie, Japan	221	Above ground	1	0	0	119
2022/5/21	Takashima, Shiga, Japan	345	Above ground	0	1	0	44
2022/5/23	Nara, Nara, Japan	369	Above ground	1	0	0	26
2022/5/23	Nara, Nara, Japan	404	Above ground	1	0	1	137
2022/5/23	Nara, Nara, Japan	400	Above ground	1	0	1	83
2022/5/23	Nara, Nara, Japan	412	Above ground	1	0	0	49
2022/5/23	Nara, Nara, Japan	410	Above ground	1	0	1	99
2022/6/2	Takashima, Shiga, Japan	536	Above ground	0	2	0	17
2022/6/9	Sakyo-ku, Kyoto, Japan	291	Above ground	1	0	0	49
2022/6/9	Sakyo-ku, Kyoto, Japan	623	Above ground	1	0	0	58
2022/6/9	Sakyo-ku, Kyoto, Japan	641	Above ground	1	0	0	43
2022/6/9	Sakyo-ku, Kyoto, Japan	624	Above ground	1	1	0	31
2022/6/9	Sakyo-ku, Kyoto, Japan	641	Above ground	0	12	0	415
2022/6/9	Sakyo-ku, Kyoto, Japan	689	Above ground	1	0	0	23
2022/6/9	Sakyo-ku, Kyoto, Japan	661	Above ground	1	0	0	49
2022/6/17	Sakyo-ku, Kyoto, Japan	334	Above ground	1	0	0	15
2022/6/24	Takashima, Shiga, Japan	381	Above ground	1	0	0	15

Cold avoidance is the most basic first line of defence in insects^35^. It has been hypothesized that subterranean termites avoid lethal low temperatures by descending underground^36^. In other members of subterranean termites, *R. flavipes* and *Coptotermes formosanus*, workers move deeper into the soil in response to a drop in temperature^37^, and there is evidence that *R. flavipes* workers are at depths > 100 cm during the winter^36^. Termites are known to construct solid royal chambers that are typically fixed in location^38^. While there is a report of the queen migration to avoid dry conditions^39^, this study reports the first evidence that termite colonies provide kings and queens with underground winter royal chambers to avoid encountering potentially lethal temperatures. Our findings have important implications for the unique reproductive systems in *R. speratus* where natural colonies are headed by multiple secondary queens^22,28^. Having multiple queens, substantially smaller than a single primary queen, may allow for increased mobility for these queens when moving to and from the winter chambers. Social insects collectively construct a variety of nest structures through local interactions among individuals^40–42^. The survival of royals (especially the primary king in *R. speratus*) is critical to ensure the maintenance of their colony^22,28^, and termite societies are selected to protect their kings and queens from extrinsic mortality^13,14,38,43^. This is the first evidence of collective behaviour in termites used for future events, as shown by the construction of the underground royal chambers in the roots of stumps before the temperature begins to drop. Our results elucidate the diversity and complexity of collective buildings in social insects.

We also report that non-reproductive castes move underground with the royals. Their movement underground serves two possible benefits. The first is cold avoidance as reported previously^36,37^, i.e. moving underground minimizes the loss of workers that are vital for the survival of the colony. The second is serving as another layer of thermal protection. Although further studies are needed, the metabolic heat of the workers may also contribute as a buffer against a decrease in the underground nest temperature. Instead of these benefits, some workers remained on the above-ground part of the stumps and logs. This may be due to risk-taking behaviour. While staying in the above-ground chambers carries the risk of freezing to death during the winter, it also provides the benefit of being able to quickly resume activities in response to rising ambient temperatures. This will beneficial for foraging, nest repair, and defence against other colonies. The proportion of workers that remain on the surface may depend on the balance of these risks and benefits.

In addition to the cold avoidance, termite kings and queens are physiologically more tolerant to the cold than workers and soldiers (Fig. 3). The well-known physiological mechanism against low temperature in insects is the accumulation of cryoprotectant metabolites such as glycerol, carbohydrates (e.g. glucose and trehalose), and polyhydric alcohols^44–46^. In the dampwood termites, *Porotermes adamsoni* and *Stolotermes victoriensis* which live in cold regions, trehalose and unsaturated lipids are preserved as cryoprotectants^47^. Kings and queens may preferentially receive these metabolites or their precursors from workers, which enables a higher accumulation of the substances in royals. The other potential cause of caste differences in cold tolerance may be due to different symbiotic microbes. *Reticulitermes* termites are known to harbour obligate symbiotic microbes in their hindgut^48–50^, which reduces the cold tolerance in *R. flavipes* workers^51^. There is a caste difference in the abundance of gut microbes^52–55^, and kings and queens are the sole castes that lack them^52^. Thus, the difference in cold tolerance among castes is consistent with the presence or absence of the symbionts (Fig. 3). Further studies are needed to determine the proximate mechanisms responsible for the caste differences in the cold tolerance of termites. Future research is also necessary to determine the supercooling point and critical thermal minima at which termites remain active. Such knowledge will help paint a clearer picture of the foraging dynamics and colony growth in certain microhabitats.

This work demonstrates the behavioural and physiological traits to overcome the cold environment at the latitudinal limits. The identification of winter royal chamber provides basic ecological information for predicting geographic distribution and spread by climate change. Although deeper underground the temperatures are warmer during the winter, the depth of the winter royal chambers in *R. speratus* is restricted by the root depth of the host tree. This provides an explanation for why termites are distributed in areas where the yearly minimum above ground temperature is much lower than their lower lethal temperature of 50% of the population, as in Hokkaido, Japan^23,24^. The identification of the winter royal chambers also opens new avenues to develop techniques to collect termite royals for ecological studies and pest control purposes. For example, since the kings and queens were in the chambers in the logs but not in the galleries in the soil during winter, meaning we may trap them by artificially planting dead woods under the soil. *R. speratus* colonies in which the primary king is replaced by a secondary king are rarely found in the field^22,28,56^, suggesting that colonies that lose their primary king are likely to perish within a short period. Therefore, the trap technique proposed here could have potential applications in pest control. In summary, this study promotes a further understanding of seasonal phenology, long-term survivorship, and life cycle, as well as contributes to the development of pest control approaches for termites.

## Materials and methods

### Seasonality in the location of royal chambers

Decayed logs on the ground in oak/pine mixed forests in Kyoto, Nara, Hyogo, and Fukui, Japan (elevation ranging from 124 to 577 m) were examined for termite kings and queens by three investigators once a month from October 2019 to September 2020. Each sampling event lasted seven hours (± 30 min). Termite colonies with kings and secondary queens were collected, and the total number of colonies with royals found per investigating event per investigator was recorded.

To investigate the location of the kings and queens in the winter, we first searched logs with termite workers on the forest ground in Kyoto, Japan in January 2019 (Fig. 1a). Then to find the royals, we traced the tunnels leading out of the logs into the soil. Following these tunnels led us to foraging areas and eventually the chamber with royals. When a chamber with royals was found, the location and depth was recorded. The logs and ground that had been dug up were then restored to their original state.

To determine the location of royal chambers in the summer, we first searched stumps with termite workers in oak/pine mixed forests in Kyoto, Shiga, Nara, Mie, and Hyogo, Japan in May and June 2021 and 2022. The stumps were dug out and examined for termite kings and queens. When a chamber with royals was found, the location (above or below ground) was recorded. In total, 22 stumps with royals were examined. Within a week of collection, all kings and queens were extracted from the logs, and the total number of each was recorded.

### Temperature measurements in the winter royal chamber and above ground

In January 2023, the coldest month in the area (Supplementary Information), three stumps with royal chamber (Colony I–III) were dugout and temperature probes with data loggers (Thermo Recorder TR-71wb, T&D Corp., Tokyo, Japan; temperature accuracy of ± 0.3 °C from − 20 to 80 °C) were placed in the underground royal chamber and the chamber in logs above the ground. The air temperature near the ground surface were also recorded at the site (Fig. 2d). Then, the stumps were returned and buried at the original place and depth. Microclimate temperatures were recorded at 1-h sampling frequencies.

### Determining lower lethal temperatures in each caste

To prepare winter-acclimatized termites, we collected 25 logs containing kings and secondary queens in Kyoto, Shiga, and Osaka, Japan, from May to September 2018–2021. The nests were kept at 25 °C from May to September, 20 °C during November, 15 °C during December, 10 °C during January, and 5 °C from February to March. Then in April, to simulate field conditions in spring we set the temperature to 20 °C. After exposure to the temperature fluctuations, all termites were extracted from logs in March 2022 and kept at 5 °C then exposed to different temperatures to test for lower lethal limits. Termites were individually placed in a 0.6 µL tube and exposed to one of five test temperatures ranging from − 10 to − 2 °C for 2 h in an incubator LTE-510 (EYELA, Japan). The temperature dropped at a rate of 1 °C min^−1^. For each test temperature, five secondary queens, five soldiers, and 20 workers were randomly selected from each colony (*N* = 25 colonies) and used in the experiment. Since a colony of *R. speratus* typically has one primary king^22,28^, five kings from five colonies were used for each test temperature. Then, termites were transferred into dishes (ca. 30 mm) with a moist unwoven cloth and kept at 25 °C. An individual was defined as dead when it had stopped moving or walking 24 h after the transfer.

### Obtaining temperature records for Kyoto city

To assess the risk of mortality due to low temperatures when overwintering above the ground, we also obtained air temperature data from the Japan Meteorological Agency^32^. Termites have a long lifespan and generation time^14,28^, meaning that long-time-scale data are needed to understand the evolution of their cold tolerance, so yearly lowest temperature records in Kyoto city for the past 140 years were used for analysis.

### Statistical analysis

The number of colonies collected in one day of field surveying was compared between different months throughout the surveying period using generalized linear mixed models (GLMMs) followed by a Tukey multiple comparison test. Investigator ID was included as a random effect and month of the year as an explanatory effect. Two-tailed paired *t* tests and *F*-tests were used to compare the mean and the variance in the temperatures in the air near the ground surface, the chambers in the above ground logs, and the underground royal chambers. *P*-values were corrected by Bonferroni methods. The non-linear effect of temperature on the survival of each caste was determined using generalized linear models (GLMs) with a binomial distribution and a logit link function. The binomial objective variable was the survival rate (the number of surviving subjects given the total number of subjects as the trial number), colony ID was included as a random effect to account for repeated measures, and test temperature as an explanatory effect. The lower lethal temperature of 50% of the population was identified from the fitted logistic regression. For comparison of cold tolerance among castes, GLM pairwise comparisons with a Holm correction were applied. The binomial objective variable was the survival rate (the number of surviving subjects given the total number of subjects as the trial number), colony ID was included as a random effect to account for repeated measures, and caste and test temperature as explanatory effects. *P*-values were calculated using the likelihood ratio test. A significance value of *P* < 0.05 was considered to indicate statistical significance. All analyses were performed using R v3.5.2 software^57^.

## Supplementary Information

Supplementary Information 1.

Supplementary Information 2.

## Data Availability

The dataset supporting the conclusions of this article is included within the article and its additional file.
