# Supplementary material for: Discovery of an underground chamber to protect kings and queens during winter in temperate termites
Source: Sci Rep. 2023 May 31;13:8809. doi: 10.1038/s41598-023-36035-1 (PMC10232455; doi:10.1038/s41598-023-36035-1)
Supplement: Supplementary file 2 — Supplementary Information 2. [file 41598_2023_36035_MOESM2_ESM.pdf]

Supplementary Information for

Discovery of an underground chamber to protect kings and queens during winter in temperate termites

Mamoru Takata, Takao Konishi, Shuya Nagai, Yao Wu, Tomonari Nozaki, Eisuke Tasaki, Kenji Matsuura

Laboratory of Insect Ecology, Graduate School of Agriculture, Kyoto University

Kitashirakawa Oiwakecho, Kyoto 606-8502, JAPAN

Supplementary Methods

Temperature measurements in the royal chamber and the ground surface during winter

Temperature measurements in the winter royal chamber and the ground surface were performed on three stumps with termite nests in Kyoto, Japan from November 2021 to May 2022. Temperature probes of loggers (Thermo Recorder TR-71wb, T&D Corp., Tokyo, Japan; temperature accuracy of ± 0.3 °C from –20 to 80 °C) were placed in the underground royal chamber and in the air near the ground surface. Then, the stumps were returned and buried at the original place and depth. Microclimate temperatures were recorded at 1-hour sampling frequencies.

Statistical analysis

Two-tailed paired t-tests and F-tests were used to compare the mean and the variance in the temperature between the royal chamber and the ground surface each month. A significance value of $P < 0.05$ was considered to indicate statistical significance. All analyses were performed using R v3.5.2 software[1].

Supplementary Results

The temperature in the winter royal chambers were on average 3.4, 4.7, 4.8, 3.6 and 0.8 °C higher than the ground surface in November (Fig. S1, two-tailed paired t-test, $t = -51.076$, $df = 2159$, $P < 0.001$), December (two-tailed paired t-test, $t = -78.235$, $df = 2231$, $P < 0.001$), January (two-tailed paired t-

test, $t = -99.273$, $df = 2231$, $P < 0.001$), February (two-tailed paired t-test, $t = -51.974$, $df = 2015$, $P < 0.001$), March (two-tailed paired t-test, $t = -11.112$, $df = 2231$, $P < 0.001$), respectively. The temperature was significantly more stable in the royal chamber than the ground in all the months surveying took place (November: F-test, $F_{2159, 2159} = 6.579$, $P < 0.001$, December: F-test, $F_{2231, 2231} = 7.579$, $P < 0.001$, January: F-test, $F_{2231, 2231} = 10.082$, $P < 0.001$, February: F-test, $F_{2015, 2015} = 38.124$, $P < 0.001$, March: F-test, $F_{2231, 2231} = 9.343$, $P < 0.001$). The few outliers recorded on the ground surface were due to the days with unusually high maximum temperatures.

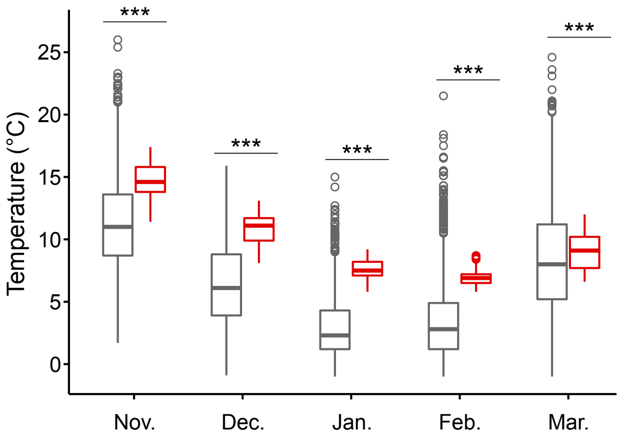

Supplementary Figure S1. Comparison of temperatures between the air near the ground surface and underground royal chambers in each month in winter from 2021−2022. Temperature data were recorded hourly in three colonies. The temperature in the air near the ground surface and underground royal chamber are shown in grey and red, respectively. ***$P < 0.001$, two-tailed paired t-tests.

Supplemental references

1. R Core Team. *R: A language and environment for statistical computing.* (R Foundation for Statistical Computing, 2018).
